# Emerging targets for cancer treatment: S100A9/RAGE

**DOI:** 10.1016/j.esmoop.2022.100751

**Published:** 2023-01-16

**Authors:** M. Valiente, J.M. Sepúlveda, A. Pérez

**Affiliations:** 1 Brain Metastasis Group, CNIO, Madrid; 2 Neuro-Oncology Unit, Hospital Universitario 12 de Octubre, Madrid; 3 Instituto de Investigación Sanitaria Hospital 12 de Octubre (imas12), Madrid; 4 Servicio de Neurocirugía, Hospital Universitario 12 de Octubre, Madrid; 5 Departamento de Cirugía, Facultad de Medicina, Universidad Complutense de Madrid, Madrid, Spain

**Keywords:** S100A9, RAGE, NF-κB, JunB, brain metastasis, radioresistance, observational prospective study, clinical trial with RAGE inhibitor

## Abstract

Developing better treatments that work for the majority of patients with brain metastasis (BM) is highly necessary. Complementarily, avoiding those therapeutic procedures that will not benefit a specific patient is also very relevant. In general, existing therapies for patients with BM could be improved in terms of molecular stratification and therapeutic efficacy. By questioning the benefit of whole brain radiotherapy as provided nowadays and the lack of biomarkers detecting radioresistance, we identified S100A9 and receptor for advanced glycation end-products (RAGE) as a liquid biopsy biomarker and a potential target for a radiosensitizer, respectively. Both of them are being clinically tested as part of the first comprehensive molecular strategy to personalized radiotherapy in BM.

## Highlights

• S100A9 binding to RAGE in cancer cells activates radioresistance in BM.
• S100A9 is a biomarker detectable in liquid biopsy.
• RAGE inhibition radiosensitizes BMs.
• A prospective multicentric observational study is evaluating S100A9 to stratify patients for radiotherapy.
• A multicentric phase I/II clinical trial will test azeliragon in combination with radiotherapy in brain tumors.

## Biological background

The contribution of the microenvironment to brain metastasis (BM) has been broadly recognized. We recently demonstrated its contribution to the induction of radioresistance in BM.^1^ Cancer cells showed high sensitivity to radiotherapy *in vitro* while turning into fully radioresistant metastases *in vivo*. The microenvironment initiates this phenotype by responding to the presence of colonizing metastatic cells with the production of cytokines that induce *S100A9* expression in the cancer cells. S100A9 produced by metastatic cells *in situ* is secreted to the extracellular space. Irradiation of metastatic cells in the brain through a yet-to-be-discovered mechanism increases receptor for advanced glycation end-products (RAGE) expression in the cancer cells. Given that the binding of S100A9 to RAGE in the cancer cell is the trigger to the activation of nuclear factor kappa B (NF-κB) signaling and JunB, which underlies the resistance mechanism to radiotherapy, this therapeutic approach paradoxically desensitizes metastatic cells to it. Although NF-κB has been reported as a general mechanism of therapeutic resistance,^2^ the exact link between S100A9 and radioresistance remains to be defined. Interestingly, unbiased profiling of experimental BMs with scRNAseq uncovered that, beyond a broad representation of detectable levels of *S100A9* among several cancer cell clusters, a specific one characterized by its stem-like properties showed the highest levels of expression. Such connection between S100A9, cancer stem cells and therapeutic resistance might involve an underlying plasticity highly influenced by the microenvironment.

## Observational study on S100A9 as a biomarker for response to radiotherapy

Our previous study has demonstrated how S100A9 plays a central role in the radioresistance of metastatic cells in the brain.^1^ In the same publication, we corroborated these findings using patient-derived organotypic cultures (PDOCs), and a retrospective analysis of patients from different institutions found an association between the presence of this biomarker in blood and local progression-free survival after surgery and radiotherapy.

As promising as these findings are, limitations still exist such as the possibility that metastatic lines used in the experiments may not be representative of the behavior of many of the tumors we may encounter in the clinical setting. Indeed, none of the melanoma experimental models tested reproduced S100A9 levels seen in a subgroup of patients with melanoma BM. However, the possibility of using PDOCs is a good alternative to circumvent this issue as they precisely mimic not only human cancer cells directly derived from the patient but also many of the conditions that the tumor encounters in the living brain. However, they also have obvious limitations such as their limited life span or their isolation from systemic circulation, which limit the sustained access of immune components. In addition, PDOCs are obtained from selected patients with good enough prognosis to be candidates for surgical treatment but at the same time present lesions deemed unsuitable for systemic treatment or isolated radiotherapy, which could also incorporate biases. Finally, the study showing the association of S100A9 levels in peripheral blood with the local control of the disease was retrospective and could not be controlled for other clinically relevant variables.

Given the secretory nature of S100A9 and the possibility of its detection in peripheral blood, we postulated it as a potential biomarker of radioresistance with significant predictive value for response and therapeutic potential given the existence of drugs blocking the downstream pathway. Thus, it is necessary to run a prospective clinical study to confirm its correlation with resistance to radiotherapy while, as far as possible, controlling for other factors that may condition the response of metastatic disease in the central nervous system or systemically.

**Figure 1:**
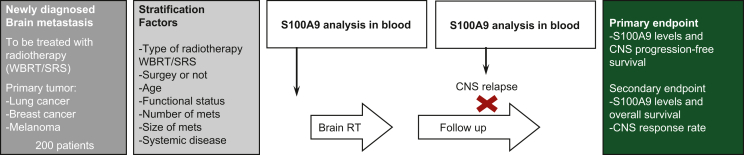
Flow chart of the prospective observational study in patients with brain metastases sponsored by RENACER. CNS, central nervous system; RT, radiotherapy; SRS, stereotactic radiosurgery; WBRT, whole brain radiotherapy.

Consequently, we have designed a prospective observational study in patients with BMs from lung cancer, breast cancer and melanoma treated with radiotherapy (Figure 1). This study will be conducted largely through RENACER (the Spanish National Network of Brain Metastases), an important collaborative effort that brings together clinical and basic researchers from 10 Spanish hospitals and the CNIO (Spanish National Cancer Research Center). RENACER is open to other centers and researchers with interest in BM.

The prospective study will analyze the presence of S100A9 in a blood sample taken at the start of radiotherapy and will be correlated with the response to treatment. Patients will be stratified depending on whether they receive holocranial radiotherapy [whole brain radiotherapy (WBRT)] or other focal radiotherapy modalities. To control for variables that might influence the levels of the biomarker, additional information such as the number of metastases, their size and whether the lesion is surgically resected will be collected. Additional variables that will be registered are relevant molecular alterations, the presence of extracranial metastases, the status of the primary tumor and the age and functional status of the patient. Our first objective is to analyze the correlation of the biomarker with local disease control and will be measured by progression-free survival in the central nervous system. The secondary objective is overall survival (censoring those cases that die due to systemic disease progression, following the recommendations of the RANO Brain Metastasis Working Group^3^). Although it might be of interest, the evaluation of cognitive function or quality of life is out of the scope of the objectives of the described protocol, which simply aims to confirm whether radiotherapy effectively targets metastatic lesions according to a biomarker.

If we can demonstrate the prediction of response to radiotherapy based on the presence of S100A9, a solid basis will be established for prognostic stratification of patients with BM as candidates for radiotherapy. Furthermore, it will strongly encourage the development of clinical trials with drugs that block the effector pathway triggered by this molecule.

## Clinical trial on RAGE inhibitor as a radiosensitizer

Taking into account the evidences of S100A9 as a mediator of resistance of BMs to radiotherapy,^1^ the inhibition of the S100A9–RAGE axis could be a novel approach to restore the sensitivity to this treatment in cancer patients with brain dissemination of the tumor. In fact, the RAGE inhibitor FPS-ZM1 was effective in reestablishing the sensitivity to radiation therapy in experimental models of BM.^1^

Following these discoveries, a new avenue of treatment opens up, using RAGE inhibitors as radiosensitizers. In this regard, it is important to note that RAGE knockout mice are healthy and develop normally,^4^ so this approach can be quite safe. Radiosensitizers are not only useful for improving the antitumor efficacy of radiotherapy, but they could also be able to reduce the radiation dose to be administered, thus improving the tolerance of the brain to radiotherapy.

In addition to the biological mechanisms explained in the previous sections related to S100A9, other RAGE ligands can also play an important role in the development of BM. S100 proteins and high-mobility group box 1 (HMGB1) are expressed and secreted by different tumor cells. These ligands interact in an autocrine manner to activate cancer cells, stimulating metastasis, proliferation, invasion and resistance to treatments.^5^ Following radiation therapy, the release of S100B and HMGB1 from injured brain parenchyma or tumor cells may activate downstream RAGE-mediated inflammatory pathways as occurs in stroke and trauma.^6^ Although the role of RAGE in radiation-associated neuro-inflammation has not been studied, RAGE activation after radiation could be extrapolated from other models of brain damage,^7^ suggesting the possibility that RAGE inhibitors may reduce brain edema after radiotherapy.

Also, from a neuro-inflammatory point of view, interleukin 6 (IL-6) has been recently identified as a pro-metastatic factor in a preclinical model of non-small-cell lung cancer BM by inducing anti-inflammatory microglia signaling via the Janus kinase 2/signal transducer and activator of transcription 3 pathway.^8^ In this regard, patients with lower IL-6 levels had better lung cancer survival rates.^8^ RAGE inhibition with azeliragon in patients with Alzheimer’s disease showed a prominent reduction in plasma levels of IL-6 as well as other cytokines.^9^

Although the role of RAGE in BMs has been studied in more depth, it may also be important in glioblastomas. In fact, HMGB1, released from necrotic glioma cells, has been implicated in glioma formation, invasion and progression through mitogen-activated protein kinase/extracellular signal-regulated kinase pathway activation.^10^ Another mechanism of gliomagenesis related to RAGE is the overexpression of S100B, a brain-specific protein for which RAGE is the receptor. S100B overexpression in gliomas enhances infiltration of tumor-associated macrophages that are mainly immunosuppressive in these tumors.^11^ IL-6 has been involved in the resistance of glioblastomas to radiotherapy.^12^ In this regard, as mentioned above, azeliragon was able to reduce IL-6 levels in patients with Alzheimer’s disease.

With this meaningful rationale, RAGE inhibitors are in development in different diseases as summarized in Table 1.

**Table 1:** RAGE inhibitors under development

Name of the compound	Mechanism of action	Phase of clinical trial development	Reference/company
Azeliragon (TTP488)	Targets RAGE V domain	-Comparative trials in Alzheimer’s diesease: negative -Phase I in association with brain radiotherapy	^9^
GM-1111	Targets V, C1, C2 RAGE domains	Mouse model	^16^
FPS-ZM1	Targets V RAGE domain	Glioma cells	^17^
Alagebrium	AGE cross-link breaker	Phase III trial in cardiovascular disorders: results not conclusive	^14^

AGE, advanced glycation end-products; RAGE, receptor for advanced glycation end-products.

There are currently no ongoing clinical trials with these drugs in cancer, but comparative studies have already been carried out in Alzheimer’s disease and cardiovascular diseases. Although the studies in Alzheimer’s disease were negative and in cardiovascular pathology the results are not definitive, important conclusions have been drawn, such as that these drugs are safe, with few reported side-effects, and that they do have biological effects, as we will see below.

**Figure 2:**
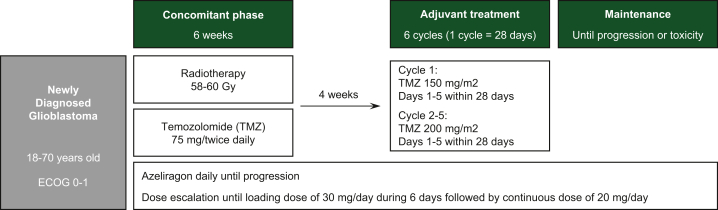
Flow chart with the design of the trial CAN-201: a phase I/II open-label study to assess safety and preliminary evidence of a therapeutic effect of azeliragon combined with conventional concurrent radiation and temozolomide in patients with newly diagnosed glioblastoma. ECOG, Eastern Cooperative Oncology Group.

**Figure 3:**
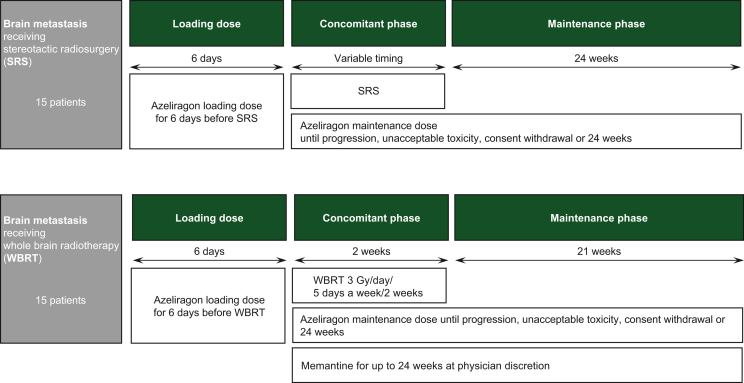
Flow chart with the design of a clinical trial with azeliragon in combination with radiotherapy in patients with brain metastases from non-small-cell lung cancer, breast cancer or melanoma.

Azeliragon (TTP488 and PF-04494700; chemical name: 3-[4-[2-butyl-1-[4-(4-chlorophenoxy)phenyl]imidazol-4-yl]phenoxy]-N,N-diethylpropan-1-amine) is an orally administered small molecule that crosses the blood–brain barrier and binds with multiple ligands, such as β-amyloid, HMGB1, S100B and advanced glycation end-products (AGEs).^13^ Azeliragon failed to show improvement in cognitive functions in Alzheimer’s disease in comparative clinical trials in which >1500 patients were dosed for up to 18 months. However, azeliragon was safe and able to significantly reduce circulating IL-6 in patients. Currently, a phase I trial to assess the safety of azeliragon combined with radiotherapy and temozolomide in newly diagnosed glioblastoma (Figure 2) is under evaluation by ethical committees. Another trial, a phase II study combining azeliragon with brain radiotherapy in BMs is being designed (Figure 3). The objective of this second study will be to evaluate whether RAGE inhibition improves the efficacy of radiotherapy in BM from various cancers. Both trials include an extensive transcriptional study.

Similar inhibitors tested include alagebrium (ALT7-11), which is an AGE cross-link breaker that reduced the atherosclerotic plaque formation in humans.^14^ Additionally, other RAGE inhibitors are also of interest, which by blocking the interaction between the RAGE V domain and β-amyloid reduced primary tumor growth, inhibition of angiogenesis and reduction of metastasis to the lungs and liver in breast cancer preclinical models.^15^

## Conclusions

• Study of experimental BM *in situ* offers novel vulnerabilities due to the influence of the microenvironment.
• S100A9 was validated in a retrospective study as a biomarker of resistance to WBRT compatible with liquid biopsy.
• A multicentric observational clinical study is ongoing within the Spanish National Network of Brain Metastasis (RENACER) to evaluate the predictive value of the biomarker.
• Blockade of RAGE prevents S100A9 induction of radioresistance in multiple experimental models of BMs sensitizing the tumor but without detectable toxicity.
• A phase II multicentric clinical trial to use RAGE inhibitor azeliragon will test safety when combined with radiotherapy in glioblastoma.
• Upon completion of the clinical trial, additional trials are planned including BMs.

## Funding

None declared.

## Disclosure

The authors have declared no conflicts of interest.
